# Vertical Jump Kinetic Parameters on Sand and Rigid Surfaces in Young Female Volleyball Players with a Combined Background in Indoor and Beach Volleyball

**DOI:** 10.3390/jfmk8030115

**Published:** 2023-08-10

**Authors:** George Giatsis, Vassilios Panoutsakopoulos, Christina Frese, Iraklis A. Kollias

**Affiliations:** 1 Biomechanics Laboratory, School of Physical Education and Sport Science at Thessaloniki, Aristotle University of Thessaloniki, 54124 Thessaloniki, Greece; bpanouts@phed.auth.gr (V.P.); hkollias@phed.auth.gr (I.A.K.); 2 Department for Biomechanics and Sportbiology, Institute of Sport and Movement Science, University Stuttgart, Allmandring 28 A, Vaihingen, 70569 Stuttgart, Germany; christina.frese@inspo.uni-stuttgart.de

**Keywords:** biomechanical analysis, kinetics, kinematics, stretch-shortening cycle, vertical jumping, surface stability, gender differences, drop jump

## Abstract

Little is known about the differences in vertical jump biomechanics executed on rigid (RJS) and sand (SJS) surfaces in female indoor and beach volleyball players. Eleven young female beach volleyball players with a combined indoor and beach volleyball sport background performed squat jumps, countermovement jumps with and without an arm swing, and drop jumps from 40 cm on a RJS (force plate) and SJS (sand pit attached to the force plate). The results of the 2 (surface) × 4 (vertical jump test) repeated-measure ANOVA revealed a significant (*p* < 0.05) main effect of the surface and the vertical jump test on the jump height and time to achieve peak vertical body center of mass velocity. A significant (*p* < 0.05) main effect of the test, but not of the surface (*p* > 0.05), was observed for the other examined biomechanical parameters. The only significant (*p* < 0.05) jump height gain difference between RJS and SJS was observed for the utilization of the stretch-shortening cycle, which was higher in SJS (15.4%) compared to RJS (7.5%). In conclusion, as the testing was conducted during the beach volleyball competitive season, the examined female players showed adaptations relating the effective utilization of the pre-stretch and enhanced stability during the execution of the vertical jump tests on a SJS compared to RJS.

## 1. Introduction

Vertical jump tests are widely considered diagnostic conditioning tests for volleyball and beach volleyball (BV) players [1,2,3,4,5] since most jumps performed in both sports are executed with countermovement and an arm swing [6]. In specific, the countermovement jump (CMJ) is observed during the execution of blocks, standing jump float serve and special counterattack actions [7]. 

The most common diagnostic vertical jump tests are the squat jump (SQJ), CMJ and drop jump (DJ), providing different information about physical fitness. Kinetic parameters, such as force, power and work, among others, as well as their respective time curves in each jump test, evaluate specific strength and conditioning capabilities. For example, a SQJ is considered an appropriate evaluation tool of the concentric muscular strength application capability [8]. As for the CMJ without an arm swing (CMJA), the effectiveness of the pre-stretch that occurs during the stretch-shortening cycle (SCC) is evaluated [9], while a CMJ with an arm swing (CMJF) tests the ability to utilize the proximal-to-distal energy flow generated from the work produced at the shoulders [10]. Finally, a DJ is used to check the ability to effectively use the SSC in a pre-stretch of great extent [11]. In addition, the difference in jump height (h_JUMP_) between a SQJ and CMJA is widely considered to represent the gain resulted from the SSC [9], and the respective gain between CMJA and CMJF is suggested to represent the upper and lower limb intra-segmental neuromuscular coordination [10,12,13]. Finally, the gain in h_JUMP_ between SQJ and DJ evaluates the effect of a greater pre-stretch on jumping ability [14]. The examination of the kinetic and temporal parameters among the different vertical jump tests is considered to provide useful insight into the neuromuscular mechanisms responsible for the optimization of jumping performance [10,12,13,14].

A vast amount of information on the decreased h_JUMP_ on sand (SJS) compared to a rigid (RJS) jumping surface exists in the literature for BV players [2,15,16,17,18,19,20,21]. The decreased h_JUMP_ on a SJS compared to RJS is associated with a lower force and power outputs [2,17,18,19] due to the less stiff surface and larger friction compared to indoor sport surfaces [15]. This deprives practitioners of applying force fast during the jumping tests, resulting in a lower power output and eventually poor jumping performance [17,18,19]. However, volleyball-specific training on a SJS during the indoor volleyball off-season resulted in higher physical fitness, such as higher endurance of quadriceps and calf muscles [22], as well as in higher jump height in SQJ and CMJ on both surfaces [22], and in the spike jump on a RJS [23]. Furthermore, there is evidence that CMJs on a RJS are not only useful to gain information regarding performance on a SJS, but also in relation to diagnosing neuromuscular imbalances in players with a mixed indoor volleyball and BV sport background for the spike jump on a SJS [16].

Previous literature has shown that game patterns [24] are gender-specific, whereby female players have slower attack tempos, but use more placed attacks and play longer rallies. Furthermore, men jump higher than women in the spike jump [25], which probably results from a combination of higher strength and power generation capabilities [26,27,28]. Furthermore, it can be also a result of different movement characteristics such as approach speed, torso incline, use of arm swing [25,28], plant angle of the dominant limb and neuromuscular activation in spike [25]. Differences in power generation capabilities could be the reason for higher kinetic parameters in CMJ despite the fact that the maximal rate of force development was even for both genders [29,30]; however, it does not explain the higher loss of jump height on a SJS compared to a RJS for women (−13%) compared to men (−9.4%) [31]. As such, it is worth noting that, although vertical jump biomechanics on a SJS have been extensively reported for male BV players, such information is missing for female BV players. With respect to the SJS, to the best of our knowledge, the only available information is that vertical jump performance in female BV players is rather constant, regardless of the sand surface [32,33], but the h_JUMP_ of the spike jump on a SJS was lower compared to a RJS [33,34].

To conclude, the respective literature lacks evidence about the modification of the jumping kinetics of female BV players when executing diagnostic vertical jump tests on a SJS, since they might have different movement characteristics than male players. The aim of the study was to investigate the possible differences in the performance and biomechanical parameters of common diagnostic vertical jump tests executed on a RJS and SJS in young female volleyball players. It was hypothesized that vertical jumps on a SJS will result in a decreased h_JUMP_ and performance gains, as well as a lower force and power outputs compared to those on a RJS.

## 2. Materials and Methods

### 2.1. Participants

The research was conducted following the requirements of the Declaration of Helsinki and the Research Ethics Code of the Aristotle University of Thessaloniki after obtaining ethical approval from the Institutional Ethics Committee (approval no.: 87/2021). A convenience sample comprising 13 young female BV players (20.2 ± 3.2 years, 1.72 ± 0.05 m, 62.9 ± 3.9 kg) was selected for examination. The participants needed to have experience on both the RJS and SJS. Participation was voluntary and was granted after obtaining a signed consent form. Of the recruited players, seven were members of the national team, with four of them having participated in major international competitions, four being national-level players and two varsity-level players.

At the time of testing, all players were participating in the competitive BV season. The inclusion criteria were participation in official BV tournaments within the previous five years, systematic participation in their training and competition BV schedule, and having been systematically (>10 h/wk) trained in indoor volleyball during the past winter. The exclusion criterion was having sustained an injury that prevented them from competition within the 6 months before the study.

### 2.2. Procedure

Basic anthropometric measures (body height, body mass) were acquired using a SECA 220 (Seca Deutschland, Hamburg, Germany) stadiometer and a Delmac PS400L (Delmac Instrumetns S.A., Athens, Greece) electronic scale. An 817E Monark Exercise Cycle (Monark-Crescent AB, Varberg, Sweden) was used for warm up, followed by dynamic stretching exercises and six sub-maximal vertical jumps, with a progressive increase in countermovement and intensity.

**Figure 1:**
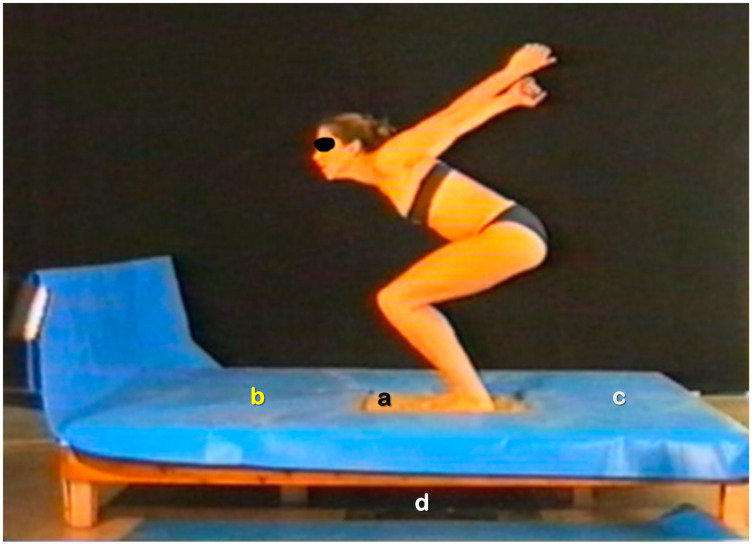
Representational depiction of the experimental set-up and execution of the countermovement jump with an arm swing on the sand jumping surface: (a) sand surface; (b) safety platform; (c) fixation points for the drop force plate; (d) force plate.

The examined vertical jump tests included an SQJ, CMJA, CMJF and a DJ from 40 cm (DJ40). Three trials were allowed for each jumping test. The intra- and inter-test resting period was 1 min and 4 min, respectively. All jumps were executed barefooted, employing procedures implemented in previous studies [17,18,19]. The surface of the force plate was considered a RJS. The vertical jump tests on a SJS were conducted on sand weighing 112.12 kg, that was contained in a wooden sand pit (Figure 1) and the depth of the sand was 0.31 m. The top-side dimensions of the wooden pit were 0.59 × 0.63 m. The bottom-side dimensions of the wooden pit were 0.46 × 0.50 m. This size was selected so that the sand pit was firmly attached to the force plate. In terms of safety, soft materials covered the edges of the wooden pit. In addition, a safety platform (1.16 × 1.50 × 0.31 m, length, width and height, respectively) surrounded the wooden pit. According to the results of a series of tests performed following the American Society for Testing and Materials (ASTM) [18], it was established that the physical properties of the sand and its grain size distribution satisfied the Federation International de Volleyball (FIVB) requirements. To avoid the compaction of sand particles during the vertical jump tests on a SJS, a tool with a 0.31 m length was used to mix and spread the sand in its entire volume within the sand pit before each trial. During data acquisition, the equality of participants’ body masses between the force plate recordings with and without the sand pit was checked.

The order of the jumping tests and the jumping surface was randomized using Matlab R2021 (The MathWorks Inc., Natick, MA, USA) scripts. In all tests, the instruction was to “jump as high as you can with the shortest push-off time”. The SQJ test initiated from a knee angle of 90° and with full foot contact on the jumping surface. If the force recordings indicated a downward movement, the trial was cancelled [17]. For the CMJ test, no restrictions were set concerning the depth of the countermovement [18]. A one-dimensional force plate (1-Dynami, ©: Biomechanics Lab AUTh, Thessaloniki, Greece) was used as the drop platform [19]. In the case of the DJ40 on the SJS, the drop force plate was fixed and adjusted within the safety platform at a height of 0.71 m. The instruction was to execute the drop with a “roll-off”, while no specific requirements were set about the depth of the countermovement during the ground contact [19].

The foot–SJS interaction was recorded with a Redlake Motionscope PCI 1S camera (Redlake Imaging Corporation, Morgan Hill, CA, USA; sampling frequency: 250 fps) to ensure that no excessive plunging into the sand occurred. This was established after the visual review of the recorded contact phase by an experienced researcher.

### 2.3. Data Acquisition and Analysis

Only the best jump in each test using the h_JUMP_ as a criterion was selected for further analysis. The criterion parameter was calculated from the vertical body center of mass (CoM) take-off velocity, which was extracted as the first-time integral of the net vertical ground reaction force (GRF) using the trapezoid rule [18]. The vertical GRF was acquired with an AMTI OR6-5-1 force plate (AMTI, Newton, MA, USA; sampling frequency: 500 Hz). GRF data recording and analysis were completed with the modules of the K-Dynami 2018 (©: Iraklis A. Kollias, Biomechanics Laboratory, Aristotle University of Thessaloniki, Thessaloniki, Greece) software. The following vertical jump biomechanical parameters were calculated using the procedures described previously [17,18,19]:

1. Temporal parameters: total push-off time (tC), time to achieve a maximum vertical GRF (tFz), time to achieve peak vertical CoM velocity (tUz) and time to achieve peak power (tP_Max_);
2. Spatial/kinematic parameters: h_JUMP_, actual drop take-off height (h_DROP_), peak CoM vertical velocity (Uz_MAX_) and maximum downward vertical CoM displacement (S_DOWN_);
3. Kinetic parameters: peak vertical GRF (Fz_MAX_), peak rate of force development (RFD) and peak power (P_MAX_).

The temporal parameters were extracted from the time curves of the respective kinetic parameters. As for h_DROP_, it was calculated with an integration of the vertical CoM velocity that was recorded from the drop force plate [19]. In turn, S_DOWN_ was extracted after integration of the vertical CoM velocity [18]. The first-time derivative of the recorded vertical GRF defined RFD, while P_MAX_ was the peak value of the multiplication product of the vertical GRF by the vertical CoM velocity during the propulsive phase [17]. Based on h_JUMP_, the following vertical jump performance parameters were also calculated [35,36]:

1. SSC gain (Equation (1)):

(1) $$\mathrm{SSC}\;\mathrm{gain}=\left(\frac{h_{\mathrm{JUMP}\left(\mathrm{CMJA}\right)}-h_{\mathrm{JUMP}\left(\mathrm{SQJ}\right)}}{h_{\mathrm{JUMP}\left(\mathrm{SQJ}\right)}}\right)\times 100$$

2. Arm swing gain (Equation (2)):

(2) $$\mathrm{Arm}\;\mathrm{Swing}\;\mathrm{gain}=\left(\frac{h_{\mathrm{JUMP}\left(\mathrm{CMJF}\right)}-h_{\mathrm{JUMP}\left(\mathrm{CMJA}\right)}}{h_{\mathrm{JUMP}\left(\mathrm{CMJA}\right)}}\right)\times 100$$

3. Drop jump gain (Equation (3)):

(3) $$\mathrm{Drop}\;\mathrm{Jump}\;\mathrm{gain}=\left(\frac{h_{\mathrm{JUMP}\left(\mathrm{DJ}40\right)}-h_{\mathrm{JUMP}\left(\mathrm{SQJ}\right)}}{h_{\mathrm{JUMP}\left(\mathrm{SQJ}\right)}}\right)\times 100$$

### 2.4. Statistical Analyses

The Shapiro–Wilk (*p* > 0.05) and the Levene tests (*p* < 0.05) were used to establish the existence of a normal distribution and equality of variance of the data, respectively. Based on the results of the above-mentioned tests, a 2 (surface: RJS vs. SJS) × 4 (jump tests: SQJ, CMJA, CMJF, DJ40) repeated-measure ANOVA with the Bonferroni adjustment was run to investigate the main effects and interaction of surface and jump modality on the biomechanical parameters of the examined vertical jumps. Significant differences were followed up with pairwise comparisons. The partial eta-squared (*η_p_*^2^) statistic was used for the determination of the effect sizes as follows: small (>0.01), medium (>0.06), and large (>0.14).

The paired sample *t*-test was used for the search of possible significant differences between the RJS and SJS relating the h_DROP_ and h_JUMP_ gain due to the SSC, the arm swing and the drop. Effect sizes were estimated based on Cohen’s *d* (≤0.49 = small, 0.50–0.79 = medium, and ≥0.80 = large effect sizes, respectively).

All statistical analyses were conducted with the level of significance set at *a* = 0.05. The IBM SPSS Statistics v.29 software (International Business Machines Corp., Armonk, NY, USA) was used for the execution of the statistical analyses.

## 3. Results

Due to the imposed inclusion and exclusion criteria, only 11 players (21.2 ± 2.3 years, 1.74 ± 0.04 m, 64.1 ± 3.5 kg) were examined. In order to reach a power of 0.8 at *a* = 0.05 with a sample size of 11 participants and 2 (surfaces) × 4 (jump types) testing, high effect sizes (0.75) are required to obtain a statistically relevant result according to the estimation made using the G*power v.3.1.9.6 software (©Franz Faul, University of Kiel, Kiel, Germany).

The results for the vertical jump biomechanical parameters are presented in Table 1. Significant (*p* < 0.05) main effects for h_JUMP_ and tUz were found between the surfaces. For both parameters, the values for the SJS condition were lower than the RJS.

In most spatio-temporal and kinetic parameters, significant (*p* < 0.05) differences among jumps, but not between surfaces, were observed. The DJ40 test was significantly different (*p* < 0.05) from the no-arm swing vertical jump tests relating the examined force parameters and power output.

Finally, no significant surface × jumping test interaction was revealed (*p* > 0.05).

No significant differences (*p* > 0.05) were revealed concerning the examined vertical jump performance parameters, except for the SSC utilization ratio, which was significantly (*p* < 0.05) higher (two-fold) on a SJS compared to RJS (Table 2). Finally, h_DROP_ was not different (*t* = 2.043, *p* = 0.068, *d* = 0.60) between the examined surfaces (34.5 ± 4.5 cm and 37.0 ± 3.8 cm for the RJS and SJS, respectively).

## 4. Discussion

The purpose of the present research was to examine the possible differences in vertical jump tests executed on RJS and SJS surfaces in young female volleyball players. The results revealed that jumping performance was lower on a SJS than RJS, but there was no difference in the examined kinetic parameters. In addition, tUz values were reached faster on a SJS compared to RJS. Furthermore, SSC gain was higher on a SJS than RJS.

In agreement with past reports [2,15,16,17,18,19,20,21,37,38], h_JUMP_ was higher on a RJS compared to SJS. Although this is not statistically relevant for all jump results yet, it is attributed to the low sample size, since only large effect sizes and not small or moderate effect sizes in a small population lead to a statically relevant result. In respect to the vertical jump tests, only h_JUMP_ on an SQJ was significantly different between the tested surfaces. This might be explained by the reported differences in the SSC gain, which could be the result of regular CMJF variation use in BV [6], such as block jumps, standing jump float serve and special counterattack actions [7], leading to better inter-segmental coordination. These adaptations have already been reported [22,39,40,41]. Since the participants were at the peak of the BV competitive period, such adaptations were most likely to occur. Biomechanical variables, such as power output, confirm the h_JUMP_ height, because no differences were observed. Similar results were reported in the past [18], but are contradictory to other previous findings [2,17].

The SJS also influenced tUz (*η_p_*^2^ = 0.433), as participants reached their peak vertical CoM velocity earlier compared to the vertical jump tests executed on a RJS. It has been suggested [14] that the generation of vertical CoM velocity is the result of the capacity of neural recruitment. A possible mechanism for this finding could be the effect of plyometric activities conducted by the players on sand that has been shown to increase the motor unit recruitment [42]. Other possible factors are the instability of the SJS, which increases the need to maintain balance. This eventually results in increased work expenditure due to the larger amount of energy absorbed [2,15,18,20,21,43,44] and decreased ability to reuse stored elastic energy during the SCC [45].

Another finding was that there was no difference in Uz_MAX_ between a SJS and RJS (*p* = 0.053). It is suggested that the Uz_MAX_ is a determinant factor for the performance differences between men and women [46]. Even though it shall not be connected with the eccentric phase of vertical jump tests [47], it is proposed that it is beneficial to achieve a higher CoM velocity during the eccentric phase [48]. This is related to increased force and power outputs at the initiation and through the entire concentric phase [49,50] that eventually result in a higher h_JUMP_ [51].

The only difference revealed for the vertical jump performance parameters was the SSC gain. The SCC gain in the present study was in reasonable agreement with past research reports [30,52]. However, our findings derived from the examined young female BV players is not in agreement with past research, suggesting that the effectiveness of SSC movements on sand rely more on the concentric rather than eccentric muscle action. This can be a result of the degradation of elastic energy resulting from the sand instability [45,53], since tC was not changed between RJS and SJS, indicating an efficient SSC function [23]. The larger SSC gain can be attributed to the fact that, as training on sand improves postural control [54], the examined young female players might have been more stable on a SJS and, thus, they optimized their jumping mechanics. This can be further supported by the fact that jump training on a SJS results in an increased CMJ jump height compared to jump training on a RJS [39].

Regarding DJ40, no drop jump gain was obtained, but rather an approximate 23% reduction in h_JUMP_. This can be attributed to a possible reduced capacity of the participants to efficiently use the SSC, since previous research on male athletes on a RJS and SJS has shown that peak angular velocity in the ankle joint when landing in a SJS is significantly lower, thus reducing the stretch mechanism [19,55]. The results for h_DROP_ confirm the above rationale, as h_DROP_ was almost two-fold from the h_JUMP_ achieved, with recommendation for the optimum h_DROP_ being in the range of 50–100% of the h_JUMP_ in CMJA for male volleyball players [56]. In contrast to previous studies on elite male BV players [19] and despite the larger RFD compared to RJS, there were no indications that the SJS led to an unstable execution of DJ40, since tFz was not different between the examined jumping surfaces. Nevertheless, it is worth noting that increased differences between a RJS and SJS compared to the other jumps were observed in DJ40 in regard to the h_JUMP_, tUz, and force parameters, especially in the RFD (+18% on SJS).

As depicted in Figure 1, the examined female BV players performed the CMJ with a full-arm swing, which is typical for volleyball players [57]. Nevertheless, a lower h_JUMP_ arm swing gain was observed in the present study when compared to professional male BV players [18], confirming previous evidence that the arm swing provides a larger h_JUMP_ gain to males than females [28]. Contrarily to the previously mentioned research, no surface effect was revealed. Past research revealed a larger range of motion in the ankle and hip joints in the CMJF compared to the CMJA on a SJS than RJS [18]. In the same study, the arm swing on a SJS was associated with a larger forward inclination of the body at the lowest position of the CoM. In general, the arm swing generates mechanical work from the musculature of the shoulder that is transferred to the lower limb muscles and eventually results in an augmented energy production for the propulsion for the jump [10,12,13]. It has been suggested that the greater upper body strength production capability in men enhances the effectiveness of this mechanism more than in women [28].

Regarding the inter-test comparison, the present study reveals that an excessive pre-stretch tension imposed by the DJ resulted in a higher force, RFD and power outcomes compared to the other jumping tests, especially those without the use of an arm swing. It has been suggested that the reflex potentiation provides additional enhancement in jumping performance [14]. This can also be attributed to the fact that SSC exercises executed on a SJS increase motor unit recruitment [42]. The comparison of the examined biomechanical parameters in vertical jump tests led to the conclusion that plyometric training aiming for a fast force application seem to improve explosiveness more effectively. However, the largest Uz_MAX_ was observed in the CMJF compared to the other jumping tests. This can be attributed to the fact that most sport-specific jumps are conducted with counter-movements and arm swings [6]. This also seems to be related to the higher P_MAX_ in the CMJF on a SJS. Nevertheless, the absence of an inter-test difference regarding tP_MAX_ can be attributed to the fact that young female volleyball players were found to rely less on fast time-depended parameters in order to maximize vertical squat jump performance [51].

We want to acknowledge some limitations of this study. First, the small sample size and homogeneity of the playing level might prevent a broad generalization of the present findings, since there are contradictory findings regarding playing level and vertical jump test performance [58,59,60], which might also be jump-specific [61]. There are some findings [60], particularly in DJ40, which show that performance is not associated with a sport-specific background rather than the ability to execute the jumping task with an optimized utilization of its kinetic factors. Second, we assumed that kinematical differences might be present between the surfaces, since the subjects did not get any instructions on the depth of the countermovement; therefore, they could self-select their movement strategy to enhance CMJ performance. Thus, a kinematical analysis would have been useful to detect such changes [50].

Future research should not only emphasize on the kinetic, but also on kinematic and electromyographic differences when jumping on rigid and sand surfaces, to examine the loading imposed on the lower limb joints and the possible modifications in the function of the neuromuscular system. The retrieved information from such studies could be applied to both performance enhancement and injury prevention. This is because in contrast to current beliefs, sand training does not necessarily involve lower kinetic parameters such as the Fz_MAX_ and P_MAX_, at least for double-legged jumps in young female players. This information might be especially important for physiotherapists working with athletes and chronic knee pain, since it likely leads to similar tendon loading compared to jumps on a RJS.

## 5. Conclusions

Young female players with a combined indoor and beach volleyball sport background performed the common diagnostic vertical jump tests on rigid and sand surfaces with no between-surface differences concerning the examined kinetic parameters. Jumping on sand resulted in: (1) a decreased jump height, especially on an SQJ; (2) a shorter time to achieve peak vertical body center of mass vertical velocity; and (3) a higher jump height gain when the countermovement was applied on the sand compared to application on a rigid surface.

The observed alterations when jumping on sand may lead to an enhanced utilization of the pre-stretch and therefore might enhance stability during the execution of the vertical jump tests. Also, the inclusion of plyometric jump training on a sand surface could stimulate the neuromuscular mechanisms that enhance jumping performance. In conclusion, the jumps performed on sand with a countermovement and arm swing or excessive pre-stretch loads imposed by drop jumps comprise jumping activities that involve favorable patterns for greater power outputs.

## Figures and Tables

**Table 1:** Mean (standard deviation) of the biomechanical vertical jump parameters on rigid (RJS) and sand (SJS) jumping surfaces (*n* = 11).

Surface	RJS					SJS					*F*	*p*	*η_p_* ^2^
Parameter	SQJ	CMJA	CMJF	DJ40		SQJ	CMJA	CMJF	DJ40		Test	Test	Test
											Surface	Surface	Surface
Body center of mass displacement													
h_JUMP_ (cm)	17.8	18.8	21.5 ^a^	13.3 ^abc^		15.1 *	17.5	20.8 ^ab^	11.8 ^abc^		40.292	<0.001	0.599
	(2.2)	(2.6)	(3.2)	(2.9)		(1.2)	(2.6)	(3.2)	(2.7)		7.566	0.007	0.085
S_DOWN_ (cm)	-	−30.9	−30.8	−36.3		-	−31.2	−30.4	−39.6 ^bc^		369.864	<0.001	0.302
		(4.2)	(4.6)	(7.0)			(2.7)	(4.1)	(7.7)		0.615	0.436	0.010
Temporal parameters													
tC (ms)	492.3	504.7	550.1	425.3 ^c^		451.9	505.8	533.7	470.9		6.843	<0.001	0.202
	(63.9)	(57.4)	(100.2)	(80.4)		(78.8)	(39.8)	(53.1)	(92.5)		0.026	0.872	0.000
tFz (%tC)	62.2	65.7	62.4	73.1		57.9	66.7	62.5	74.5 ^a^		3.964	0.011	0.128
	(17.2)	(4.7)	(17.7)	(11.5)		(17.3)	(6.5)	(17.8)	(15.4)		0.023	0.881	0.000
tUz (%tC)	75.5	75.9	76.6	71.9 ^abc^		72.8	74.9	75.9 ^a^	68.9 ^abc^		20.608	<0.001	0.433
	(1.7)	(1.9)	(2.5)	(3.2)		(1.2)	(2.0)	(3.4)	(4.3)		10.720	0.002	0.117
tP_MAX_ (%tC)	74.4	75.3	76.0	71.1		71.6	75.4	78.0	70.5		2.896	0.040	0.097
	(3.6)	(3.0)	(4.9)	(15.2)		(4.4)	(2.1)	(8.6)	(8.7)		0.052	0.821	0.001
Kinematic parameters													
Uz_MAX_ (m/s)	2.46	2.52	2.67 ^a^	2.23 ^abc^		2.36	2.46	2.65 ^ab^	2.19 ^abc^		40.550	<0.001	0.600
	(0.12)	(0.12)	(0.14)	(0.18)		(0.09)	(0.13)	(0.14)	(0.16)		3.840	0.053	0.045
Kinetic parameters													
Fz_MAX_ (N/kg)	2.06	2.27	2.28	3.27 ^abc^		2.06	2.26	2.30	2.97 ^abc^		34.827	<0.001	0.566
	(0.12)	(0.16)	(0.14)	(0.75)		(0.16)	(0.13)	(0.18)	(0.64)		0.820	0.368	0.010
RFD (kN/s)	5.1	8.5	7.3	31.5 ^abc^		5.9	9.0	7.0	38.1 *^abc^		108.053	<0.001	0.800
	(1.5)	(5.6)	(4.1)	(9.8)		(1.7)	(4.2)	(3.0)	(12.0)		2.033	0.158	0.158
P_MAX_ (W/kg)	21.4	21.5	25.6	28.7 ^b^		20.2	20.7	26.7 ^ab^	26.9 ^ab^		14.564	<0.001	0.350
	(2.7)	(3.2)	(4.3)	(8.1)		(3.2)	(3.7)	(4.3)	(3.4)		0.530	0.469	0.006

*: *p* < 0.05 vs. RJS surface; ^a^: *p* < 0.05 vs. SQJ; ^b^: *p* < 0.05 vs. CMJA; ^c^: *p* < 0.05 vs. CMJF. Abbreviations: h_JUMP_: jump height; S_DOWN_: maximum vertical downward body center of mass (CoM) displacement; tC: total push-off time; tFz: time to achieve maximum vertical ground reaction force (GRF); tUz: time to achieve maximum vertical CoM velocity; tP_MAX_: time to achieve maximum power during the upward phase; Uz_MAX_: peak vertical CoM velocity; Fz_MAX_: peak vertical GRF; RFD: peak rate of force development; P_MAX_: peak power.

**Table 2:** Mean (standard deviation) of the vertical jump performance ratios on rigid (RJS) and sand (SJS) jumping surfaces (*n* = 11).

Parameter	RJS	SJS	*MD*	*SE*	*t*	*p*	*d*
SSC gain (%)	7.5 (8.4)	15.4 (8.6)	7.9	3.6	2.516	0.031 *	0.93
Arm swing gain (%)	14.9 (9.2)	18.9 (10.7)	4.0	4.3	0.748	0.471	0.40
Drop jump gain (%)	−23.7 (12.9)	−22.3 (15.2)	1.4	6.0	0.260	0.800	0.10

*: *p* < 0.05; Abbreviations: SSC: stretch-shortening cycle: *MD*: mean difference; *SE*: standard error of the mean; *d*: Cohen’s *d*.

## Data Availability

The data that were acquired and analyzed in the present study are available from the corresponding author upon reasonable request.
